# An Orbital Pseudotumor Secondary to Systemic Lupus Erythematosus: A Case Report

**DOI:** 10.7759/cureus.44994

**Published:** 2023-09-10

**Authors:** Elham Alqahtani, Yara Albalawi, Nouf A Altwaijri, Lama Alqahtani, Shahad Alshail

**Affiliations:** 1 Ophthalmology, King Faisal Specialist Hospital and Research Centre, Riyadh, SAU; 2 Orthopedic Surgery, King Saud Medical City, Riyadh, SAU; 3 Medicine and Surgery, Alfaisal University College of Medicine, Riyadh, SAU

**Keywords:** systemic lupus erythematosus, pseudotumor orbit, ocular complications, nonspecific orbital inflammation, orbital complications

## Abstract

Nonspecific orbital inflammation (NSOI), the primary cause of painful orbitopathy mostly in adults, can either manifest as localized or diffused. Periorbital edema or swelling is the most common sign followed by proptosis. NSOI or orbital pseudomotor secondary to systemic lupus erythematosus (SLE) is very uncommon in the Kingdom of Saudi Arabia. This is the first reported case from Saudi Arabia. The patient first presented to the outpatient department during her gestational period. Her chief complaint was right eye swelling and pain when she woke up in the morning. Her past medical history was positive for irritable bowel disease and SLE. A slit lamp examination revealed chemosis with conjunctival injections in the right eye and mild temporal chemosis in the left eye. Funduscopic examination after pupillary dilation revealed hyperemic discs with venous tortuosity more prominent in the right eye. Serum albumin level was low at 29 g/L. Orbital magnetic resonance imaging without contrast showed bilateral diffuse preseptal soft tissue swelling more prominent on the right side with diffuse bilateral congestion of intraorbital fat, including intraconal and extraconal fat. There was associated fat stranding around the optic nerves bilaterally. The bilateral extraocular muscles showed a diffusely increased T2 signal compatible with edema. The patient was given a bolus of intravenous methylprednisolone for three days. She had a satisfactory recovery. Early diagnosis is important to rule out other differential diagnoses, such as orbital cellulitis and lymphoma.

## Introduction

Nonspecific orbital inflammation (NSOI) is classified as a benign inflammatory condition of the orbit with a polymorphous lymphoid infiltration and variable degrees of fibrosis with no recognized systemic or local etiology [1]. Previously, it was known as idiopathic orbital inflammation, idiopathic orbital pseudotumor, and idiopathic ocular inflammatory syndrome. NSOI typically occurs in five orbital areas or patterns [2]. The extraocular muscles (myositis), lacrimal gland (dacryoadenitis), anterior orbit (scleritis), orbital apex, or widespread inflammation across the orbit are the most frequent in that order [3]. Although symptoms vary depending on the tissue involved, deep-rooted, boring pain is a common sign. Along with eyelid erythema and soft tissue edema, extraocular muscle limitation, proptosis, conjunctival inflammation, and chemosis are frequent. Adults with simultaneous bilateral orbital irritation may have systemic vasculitis. Systemic vasculitis is any arterial or venous inflammation that can lead to a neurologic end stage. Certain infections, immune system-altering diseases, and allergic reactions are the predisposing factors for the same. It is mostly diagnosed by an antineutrophil cytoplasmic antibody test. It is an autoimmune disease leading to the narrowing or necrosis of blood vessels [4]. To our knowledge, this is the first case of orbital inflammation caused by systemic lupus erythematosus (SLE) to be documented in Saudi Arabia. The pathophysiology of NSOI is debatable, and it often responds quickly and favorably to systemic corticosteroid therapy and other immunosuppressive drugs, suggesting a cell-mediated component.

## Case presentation

A 29-year-old female, gravida 2 para 1 (G2P1+0), in her 13th week of gestation, presented to our hospital with right eye swelling and pain when she woke up in the morning. The night before, she had noticed mild swelling of her right eye associated with excessive tearing and a foreign body sensation, for which she applied lubricant eye drops. The next day, she was unable to open her right eye and developed eye pain, swelling, redness, and tearing. She was not taking any drugs before the onset of symptoms. Her past medical history was positive for irritable bowel disease and SLE, which was maintained on hydroxychloroquine 400 mg daily, prednisone 5 mg daily, and methotrexate 15 mg weekly. She did not have any history of thyroid dysfunction or orbital trauma, nor was there any history of sinusitis or rhinitis. She was known to be allergic to penicillin and enoxaparin, which caused a skin rash and dyspnea.

**Figure 1:**
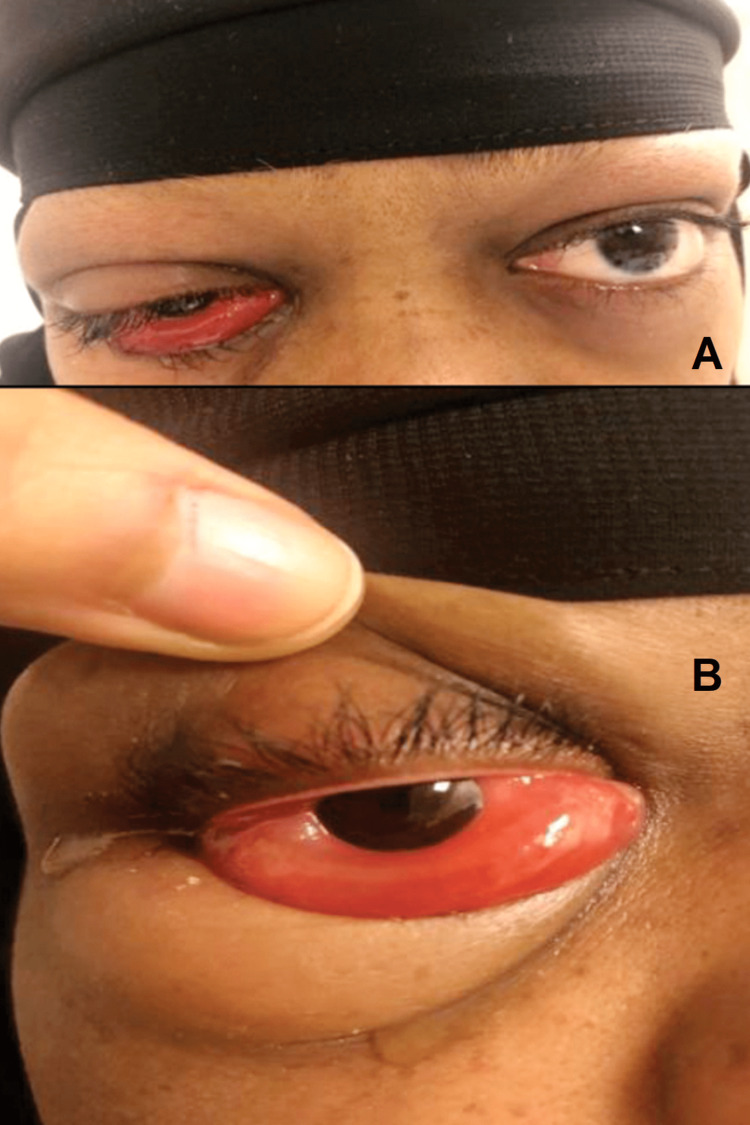
Patient’s right eye upon admission showing periorbital edema, chemosis, and limited ocular motility.

Upon examination, visual acuity was 20/20 OU, intraocular pressure (IOP) was 26 mmHg OD and 25 mmHg OS, and the relative afferent pupillary defect was negative OU. Ocular motility of the right eye was limited in all fields of gaze and exacerbated the pain without proptosis; color vision was intact. A slit lamp examination revealed grade 2 chemosis anterior to the so-called gray line in the left eye (Figures 1A, 1B). Cornea and anterior-segment examination were normal OU.

Funduscopic examination after pupillary dilation revealed a hyperemic disc with more venous tortuosity in the right eye. Complete blood count test results showed that hemoglobin was slightly low at 99 g/L, white blood cell count was 11.06 × 10^9^ cells/L, and platelets were low at 115 × 10^9^ cells/L. Blood urea was low at 4.1 mg/dL, and creatinine was also low at 0.38 mg/dL. Serum albumin level was low at 29 g/L. Urine analysis disclosed +3 proteins. All other hematological investigations, including liver and thyroid function tests, were within normal limits (Table 1).

**Table 1:** Thyroid and liver function test results.

Thyroid function tests	
Thyroid-stimulating hormone	1.2 IU/mL
T3	1 ng/mL
T4	5 ng/dL
Liver function tests	
Total bilirubin	0.8 mg/dL
Direct bilirubin	0.4 mg/dL
Alkaline phosphatase	55 IU/L
Alanine transaminase	5 IU/L
Albumin	29 g/L

**Figure 2:**
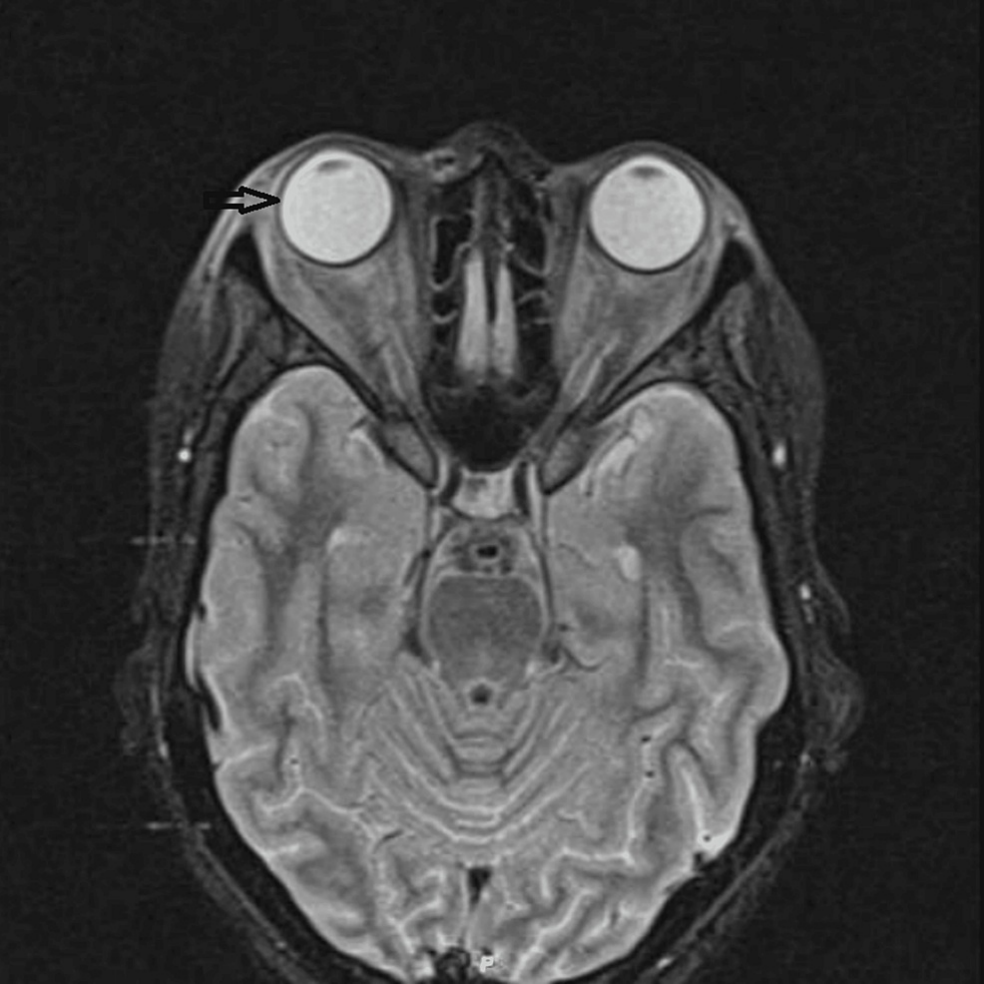
Orbital magnetic resonance imaging without contrast showing bilateral diffuse preseptal soft tissue swelling more on the right side.

**Figure 3:**
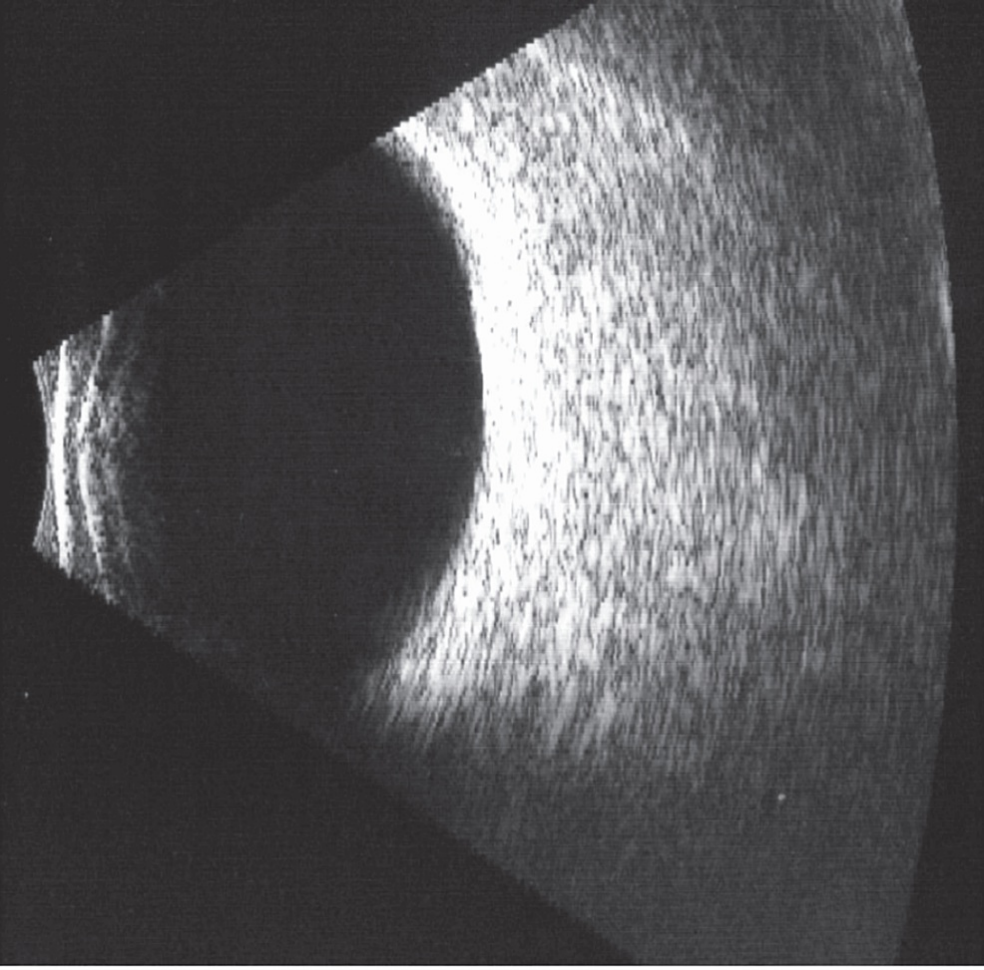
Bright scan ultrasonography of the right eye.

Orbital magnetic resonance imaging (MRI) without contrast showed bilateral diffuse preseptal soft tissue swelling more on the right side with diffuse bilateral congestion of intraorbital fat, including intraconal and extraconal fat. There was associated fat stranding around the bilateral optic nerves. The bilateral extraocular muscles show a diffusely increased T2 signal compatible with edema. The brain, ocular globes, optic nerves, and cavernous sinuses were normal. The paranasal sinus was clear (Figure 2). Additional B-scan ocular ultrasound was normal in both eyes (Figure 3).

**Figure 4:**
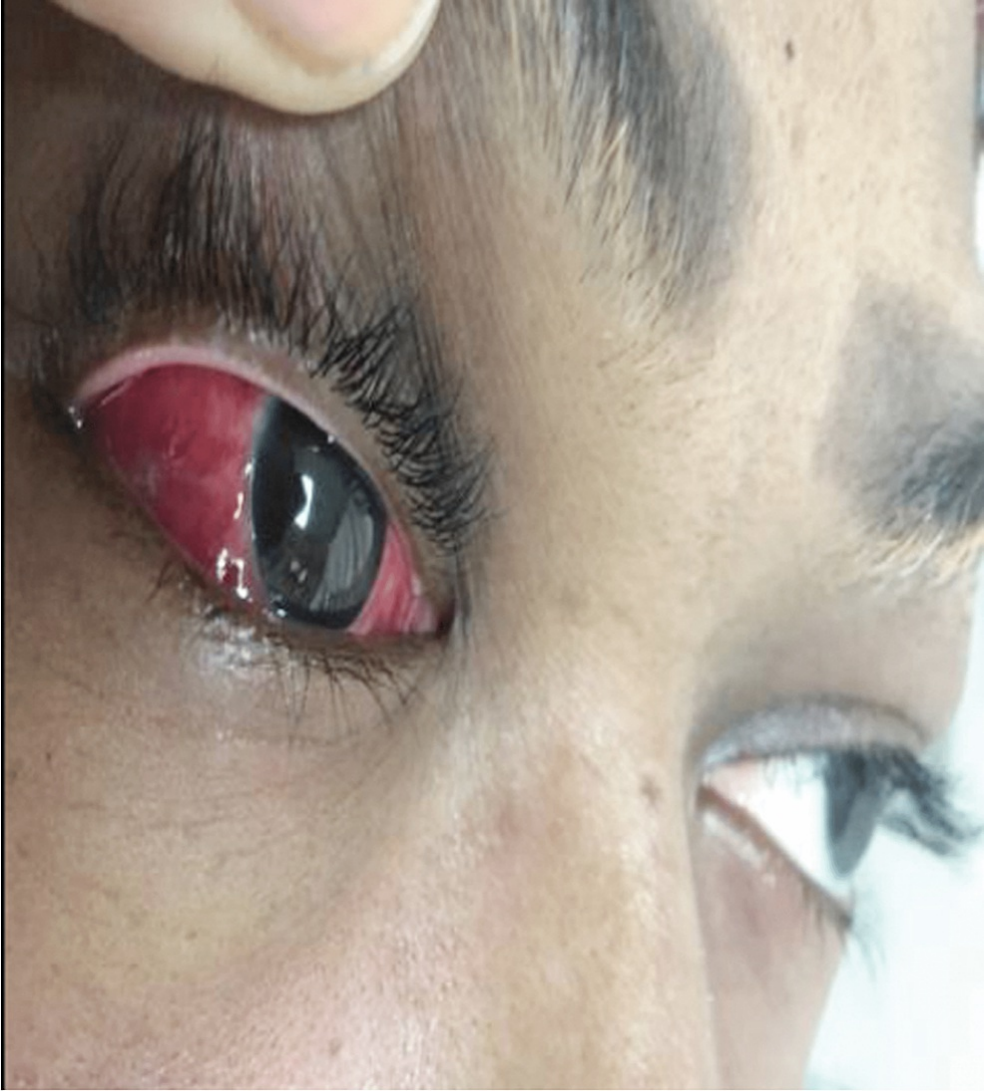
Patient’s right eye after 24 hours showing significant reduction in chemosis and improved ocular motility.

The patient was admitted, and a bolus of intravenous (IV) methylprednisolone 1 g was started and administered for three consecutive days and then tapered for weeks. Prednisolone decreases inflammation of the eye by treating swelling, redness, and itching. After 24 hours, there was a significant reduction in the degree of chemosis, less pain, and improved ocular motility (Figure 4). Visual acuity and color vision were intact with an IOP of 18 mmHg OU.

**Figure 5:**
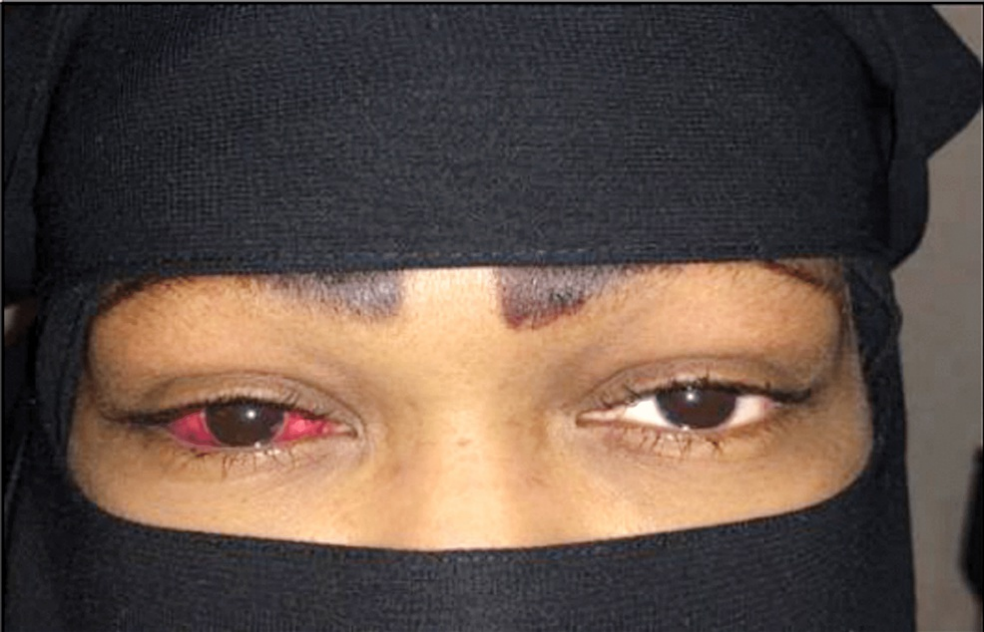
The patient’s right eye showing mild chemosis and conjunctival injections.

Three days after methylprednisone administration, the pain completely subsided with only mild chemosis and conjunctival injections in the right eye (Figure 5). She was discharged from our hospital, and the systemic corticosteroid was tapered gradually over the next four months. A follow-up appointment was scheduled at an appropriate interval.

## Discussion

Ocular complications in SLE can range from mild (e.g., keratoconjunctivitis sicca) to slightly threatening diseases (e.g., acute orbital ischemia and infarction), which need immediate intervention. Overall, one-third of orbital complications in SLE are vasculitis, myositis, and panniculitis [5,6]. Orbital manifestation is rare and can be present with extraocular muscle restriction, proptosis, orbital pain, enophthalmos, and chemosis. It is most commonly unilateral, but bilateral involvement is frequent. Immune complex deposition and circulating autoantibodies against eye antigens play a significant role in the pathogenesis of ocular disease associated with SLE [4,7]. In our case, the low albumin level resulted in severe chemosis and preorbital edema.

Early diagnosis is important to rule out other considerable differential diagnoses such as orbital cellulitis and lymphoma (Table 2). Computed tomography imaging of NSOI shows unpredictable attenuation and varying degrees of contrast enhancement. Because the clinical findings and conventional MRI findings in OIS, orbital lymphoid lesions, and orbital cellulitis often have considerable overlap, it can be difficult to make a definitive diagnosis without a pathologic specimen.

**Table 2:** Differential diagnosis of NSOI. CRP: C-reactive protein; ESR: erythrocyte sedimentation rate; NSOI: nonspecific orbital inflammation; TSH: thyroid-stimulating hormone

Type	Laterality	Onset	Pain	Differentiating findings	Associated signs and symptoms	Labs	Imaging
NSOI	Usually unilateral	Acute	Manifestation of pain	Local tenderness and eyelid swelling	Minimal symptoms	Elevated ESR and CRP	Muscle enlargement, tendon involvement
Orbital cellulitis	Unilateral	Acute	Pronounced pain	Chemosis, ptosis, erythema	Fever	Leukocytosis	Subperiosteal abscess and venous
Thyroid eye disease	Bilateral	Variable onset	Pain present	Optic neuropathy lid retraction	Goitre, fatigue, heat intolerance	Elevated T3 and T4, decreased TSH level	Increased orbital fat
Sarcoidosis	Unilateral or bilateral	Acute or subacute	Presence of pain	Mass/Swelling ptosis	Erythema nodosum, persistent cough	Decreased pulmonary function	Hilar lymphadenopathy

Therefore, it would be clinically useful to have a non-invasive method to help distinguish these processes [8]. Systemic corticosteroids are the mainstay of therapy for NSOI during the acute stage [2]. In our case, there was a favorable response to methylprednisone therapy. To our knowledge, there are only a few reported cases of orbital inflammation secondary to SLE worldwide, but there are no reported cases from Saudi Arabia.

## Conclusions

This case report describes an unusual presentation of NSOI in a pregnant patient with SLE who was maintained on hydroxychloroquine, prednisolone, and methotrexate. Based on the clinical picture and orbital MRI findings, NSOI was diagnosed. The diagnosis of NSOI is supported by a quick and good response to systemic corticosteroids. Bilateral involvement of the orbit in NSOI can suggest the possibility of systemic vasculitis, and it is important to rule out other causes of orbital inflammation, especially in patients with underlying autoimmune diseases. Ophthalmologists and rheumatologists should be aware of this association and should consider NSOI in the differential diagnosis of orbital inflammation, even in patients who are already being treated with immunosuppressive agents for autoimmune diseases. Further research is required to fully comprehend the pathophysiology of NSOI, identify relevant risk factors, and predict therapy response.
